# 751. Impact of Changing from a Three-step to Two-step Testing Algorithm for the Diagnosis of *Clostridioides difficile*

**DOI:** 10.1093/ofid/ofab466.948

**Published:** 2021-12-04

**Authors:** Jonathan Lapin, Vasilios Athans, Shawn Binkley, Tiffany Lee, Sonal Patel, Melissa Richard-Greenblatt, Laurel Glaser, Keith W Hamilton, Stephen Saw

**Affiliations:** 1 Hospital of the University of Pennsylvania, Philadelphia, PA; 2 University of Pennsylvania, Philadelphia, PA

## Abstract

**Background:**

The optimal method for laboratory diagnosis of *Clostridioides difficile* infection (CDI) remains undefined and national guidelines do not make a recommendation for a preferred diagnostic algorithm. Aiming to improve infection control measures, the Hospital of the University of Pennsylvania changed its testing process for the diagnosis of CDI from a 3-step to a 2-step algorithm (Figure 1) in September 2018. Starting an algorithm with nucleic acid amplification testing (NAAT) has been hypothesized to lead to potential diagnostic uncertainty if the result is positive by NAAT alone, as this test cannot distinguish between active infection and colonization.

Three-Step and Two-Step Diagnostic Testing Algorithms

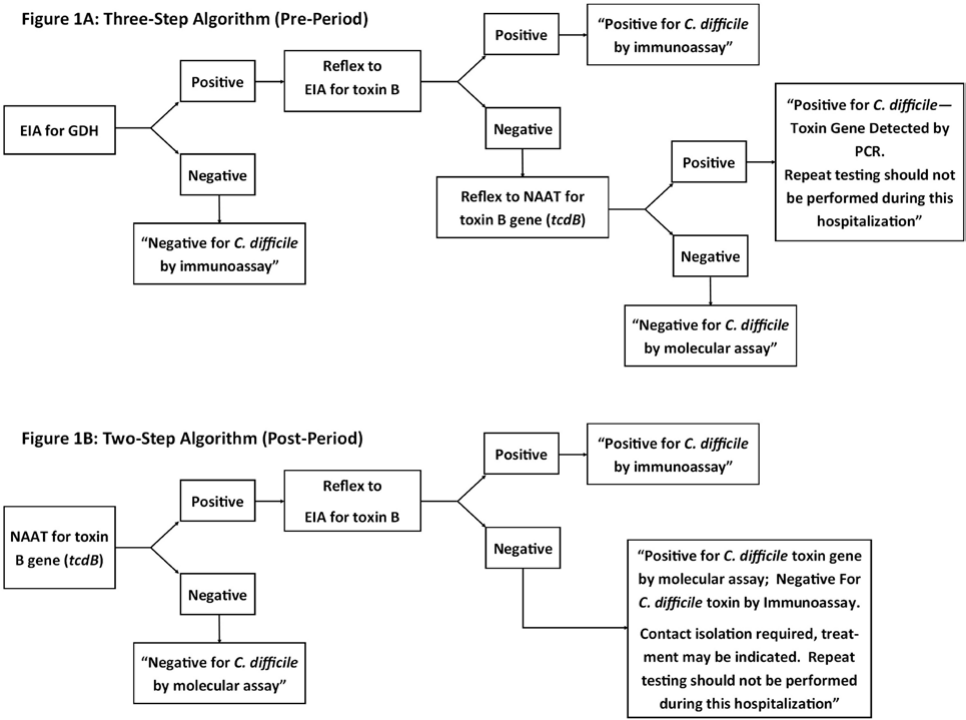

**Methods:**

This retrospective, single-center, quasi-experimental study included patients ≥ 18 years of age that tested positive for *C. difficile* between May 1^st^, 2018 and January 31^st,^ 2019. The study period encompassed 4 months prior to the algorithm change, a 1-month washout immediately following the change, and the subsequent 4 months. The primary outcome was proportion of patients who tested positive for *C. difficile* and received targeted treatment for CDI. Secondary outcomes included total number of patients who tested positive for *C. difficile* and received targeted treatment for CDI, duration of treatment for CDI, and hospital length of stay.

**Results:**

Sixty-nine patients in the pre-group (3-step) and 75 patients in the post-group (2-step) tested positive for *C. difficile.* A higher proportion of patients in the post-group tested positive by NAAT alone (59.4% vs. 73.3%). CDI severity and prior history of CDI were similar between groups. The primary outcome occurred in 89.9% of patients in the pre-group and 83.8% in the post-group (p=0.213). Sixty-two patients in each group received targeted treatment for CDI (p=0.213), median treatment duration was 15 (IQR 11.25-25.75) and 14 (IQR 11-25) days (p=0.505), and median hospital length of stay was 9 (IQR 3-15) and 6 (IQR 3-20) days (p=0.690) in the pre-group and post-group, respectively.

**Conclusion:**

Although there was a higher percentage of patients in the post-group that tested positive for *C. difficile* by NAAT alone, there was no difference in the proportion or total number of patients who received targeted CDI treatment between time periods.

**Disclosures:**

**All Authors**: No reported disclosures

